# A Finger-Actuated Sample-Dosing Capillary-Driven Microfluidic Device for Loop-Mediated Isothermal Amplification

**DOI:** 10.3390/bios14090410

**Published:** 2024-08-23

**Authors:** Xuan Le, Jianxiong Chan, James McMahon, Jessica A. Wisniewski, Anna Coldham, Tuncay Alan, Patrick Kwan

**Affiliations:** 1 Department of Neuroscience, School of Translational Medicine, Monash University, Melbourne, VIC 3004, Australia; thanh-xuan.le@monash.edu (X.L.); jianxiong.chan@monash.edu (J.C.); 2 Dynamic Micro Devices Laboratory, Department of Mechanical and Aerospace Engineering, Monash University, Clayton, VIC 3800, Australia; 3 Department of Infectious Disease, Alfred Hospital and School of Translational Medicine, Monash University, Melbourne, VIC 3004, Australia

**Keywords:** point-of-care testing, POCT, loop-mediated isothermal, LAMP, finger-actuated microfluidic, capillary-driven microfluidic

## Abstract

Loop-mediated isothermal amplification (LAMP) has attracted significant attention for rapid and accurate point-of-care diagnostics. However, integrating sample introduction, lysis, amplification, and detection steps into an easy-to-use, disposable system has so far been challenging. This has limited the uptake of the technique in practical applications. In this study, we developed a colourimetric one-step LAMP assay that combines thermolysis and LAMP reaction, to detect the SARS-CoV-2 virus in nasopharyngeal swab samples from COVID-19-infected individuals. The limit of detection was 500 copies per reaction at 65 °C for 25 min in reaction tubes. Additionally, we developed a finger-operated capillary-driven microfluidic device with selective PVA coating. This finger-actuated microfluidic device could self-dose the required sample amount for the LAMP reaction and inhibit sample evaporation. Finally, we integrated the LAMP assay into the microfluidic device by short-term pre-storage of the LAMP master mix. Using this device, nasopharyngeal swab samples from COVID-19-infected individuals showed positive results at a reaction time of 35 min at 65 °C. This integrated device may be adapted to detect other RNA viruses of interest rapidly.

## 1. Introduction

Isothermal nucleic acid amplification (ISAmp) assays are a class of alternative methods of DNA amplification that take advantage of different enzymes to target and amplify the genetic material at a single temperature [1]. Among several ISAmp assays, loop-mediated isothermal amplification (LAMP) is one of the most common techniques that has gained a huge interest in development and commercialisation. Invented by Notomi et al. in 2000 [2], LAMP utilises two or three primer pairs to target six or eight regions of the DNA strands based on the displacement activity of *Bst* DNA polymerase. LAMP has several advantages over polymerase chain reaction (PCR), such as fast reaction time, cost-effective assays, simple incubation conditions, and flexible result readout [3]. During COVID-19, many studies focused on developing reverse transcriptase-LAMP (RT-LAMP) assay for SARS-CoV-2 detection using different types of samples and combining different sample treatment methods [4]. The RT-LAMP assay could give reliable detection results with the sample having the RT-qPCR cycle threshold (Ct) number below 30 within 30 min [5].

In contrast, the RNA extraction step is crucial for nucleic acid amplification techniques (NAATs) like PCR or LAMP, which involve breaking open viral particles, releasing their RNA, and separating them from other cellular components. This step can take approximately 30 min with conventional kits [6]. The quality and quantity of extracted nucleic acid can vary depending on the sample types [7] and extraction protocols [8], which may introduce inhibitors to NAATs [4], thereby impacting the sensitivity and specificity of the molecular detection assay. Consequently, this can affect the accuracy of COVID-19 diagnosis and subsequent treatment decisions. Further, due to the complexity of molecular procedures, it is still challenging to adapt RNA extraction to the point-of-care testing (POCT) device, which prioritises simplicity, low cost, and ease of use for non-expert users.

Microfluidic technology has been widely studied in biomedical and medical diagnosis applications. This miniatured system holds several outstanding features, such as portability, reagent-saving, and compactness [9]. Microfluidic technology has a high potential to integrate with molecular procedures, including sample treatment, nucleic acid amplification, and result reveal. Passively driven microfluidics has gained more interest in applications due to the elimination of external components using different methods (surface tension, pressure-driven, etc.) [10]. Pressure-driven microfluidics, particularly finger-actuated, is one of the common types which can introduce mechanical force to the microfluidic system for operation. For example, some studies focused on designing a press valve to manipulate the fluid flow without requiring special pumps or mixers [11,12]. Without the requirement of bulky equipment, the finger-actuated microfluidic system has enhanced portability and simplicity, enabling applications in POCT devices for blood cell counting, nucleic acid amplification, and immunoassay [13]. In addition, at the microscale or below, the capillary force is inevitable and influential on fluid flow [14]. By altering the geometries [15] or surface properties [16], the capillary force can change the surface tension and can act like a valve to enhance or stop the fluid flow inside the microfluidic system.

Adaption of LAMP into the POCT device will be a promising tool to control the spread of contagious diseases, especially in low-income settings. The Lucira™ Check It COVID-19 Test kit is the first home-based RT-LAMP point-of-care testing (POCT) device that has been approved for use under the Food and Drug Administration’s emergency use authorisation during the pandemic [17]. With this POCT device, the samples would be collected by the user, following simple sample processing, and the result can be revealed after 30 min. However, this POCT is for single use only, and all the kit materials need to be disposed of after use. This causes potential pressure in waste treatment, which requires careful consideration. To address these challenges, our previous study successfully developed a reusable, ultra-portable, self-contained LAMP-based POCT device for active COVID-19 infection diagnosis [18]. The reaction chip included separate chambers for sample lysing/RNA extraction and LAMP reaction at different temperatures (95 °C and 60 °C, respectively). The lysed sample was transferred manually from the sample lysing/RNA extraction chamber to the LAMP reaction chamber using a torque-actuated valve. The results yielded within 35 min including 5 min or more of sample transfer. The time might be slightly varied in each step depending on the users. Combining the two steps of RNA extraction and amplification into a single chamber would simplify the procedures and reduce labour work. However, 95 °C is a relatively high temperature, which would require a few minutes to reach or to cool down from, resulting in a longer total time of running one sample. In addition, high temperatures may cause RNA degradation [19].

**Figure 1:**
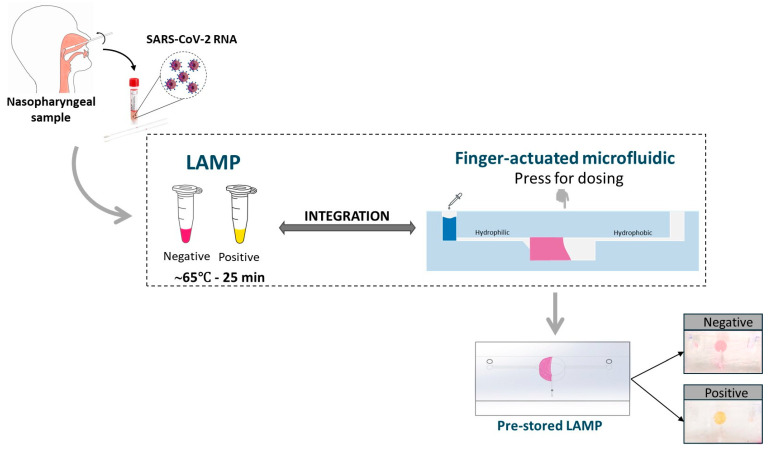
Graphical abstract of this study.

In this study, we developed a one-step LAMP reaction for RNA amplification integrated with a finger-actuated capillary-driven microfluidic (or finger-actuated microfluidic in short) chip (Figure 1). To overcome the limitations of traditional nucleic acid extraction, we incorporated the sample lysing and RNA extraction steps directly into the LAMP reaction using thermolysis of the cells, facilitated by the working temperature of the LAMP reaction. This transformed the process into a seamless one-step procedure. We further introduced a finger-actuated microfluid with a selective polyvinyl alcohol (PVA) coating that could self-dose the required sample amount and promote the visualisation of colourimetric LAMP by the naked eye. Importantly, PVA coating was used to enhance the capillary effect and reduce the polymerases and LAMP inhibitors on the microfluidic surface [20]. Given its compact design and simple operation, this microfluidic device holds promise in bringing molecular testing closer to the point of patient care.

## 2. Materials and Methods

### 2.1. LAMP Reactions Development and Optimisation

#### 2.1.1. Colourimetric LAMP Primer Design

The LAMP reaction consisted of 2X WarmStart^®^ Colourimetric LAMP Master Mix (New England Biolabs, Ipswich, MA, USA), 0.2 µM F3 and B3 primers, 1.6 µM FIP and BIP inner primers, and 0.4 µM LF and LB loop primers (desalted grade from Integrated DNA Technologies, Coralville, IA, USA). The detailed primer sequences are described in Table 1. In each reaction, 5 µL of the sample was added to achieve the final volume of 12.5 µL.

#### 2.1.2. Temperature for Thermolysis of One-Step LAMP

Positive RNA control (Twist Bioscience, San Francisco, CA, USA) was used for the assay temperature test to obtain the highest temperature for a positive reaction. The temperatures tested were 60 °C, 65 °C, and 70 °C.

#### 2.1.3. Clinical Samples

Clinical sample handling: The clinical samples were nasopharyngeal swabs taken from the COVID-19 Biobank (Department of Infectious Diseases, Alfred Health). Samples were obtained from adults with a PCR-confirmed diagnosis of COVID-19, who were enrolled to donate additional blood samples, residual transport media from viral swabs, and clinical information (Alfred Health Human Research and Ethics Committee Number 182/20). The clinical samples are stored in Universal Transport Medium^®^ UTM (Copen, Brescia, Italy) at −80 °C until use.

Limit of detection (LOD): The clinical samples with a known viral count were used in this study to achieve a concentration ranging from 200 to 3000 copies per reaction. The lowest viral concentration of SAR-CoV-2 detected by the LAMP assay with a sensitivity of 90% or more in 20 repeats was determined as the LOD.

Specificity of different clinical samples: A panel of 10 negative COVID-19 UTM nasopharyngeal samples was used to evaluate the specificity.

### 2.2. Microfluidic Design and Fabrication

**Figure 2:**
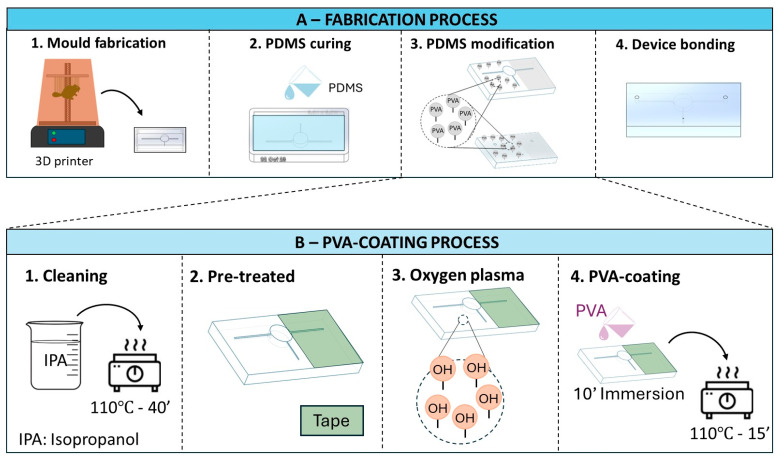
Schematic (**A**) illustrates four main steps of the fabrication process for the microfluidic devices. (**B**) describes the detailed steps of selective PVA coating for the PDMS modification step (A-3).

The complete process of making the finger-actuated capillary-driven microfluidic device is described in Figure 2 below.

#### 2.2.1. Mould Design and Microfluidic Device Fabrication

**Figure 3:**
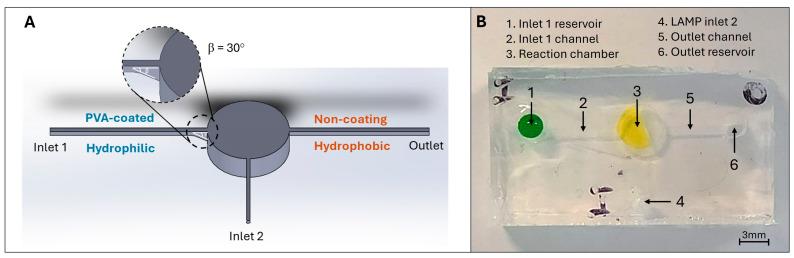
(**A**) Schematic of the finger-actuated microfluidic devices with the selective PVA-coating region. (**B**) shows the actual image of the fabricated finger-actuated microfluidic devices.

Mould designs: The microfluidic pattern was designed using the SolidWorks software 2022 (Dassault Systèmes, Vélizy-Villacoublay, France). The device consists of two inlets, one outlet, and one cylindrical reaction chamber containing 25 µL of liquid (as shown in Figure 3A). The ratio between width and height is 1 for both the inlets and outlets. The 5 mm top layer includes two holes that would match the inlet 1 and the outlet with a diameter of 3 mm.

Mould fabrication. Form 3BL printer (Formlabs Inc., Somerville, MA, USA) was used to produce the moulds. The moulds were heated at 120 °C for 1 h as suggested by Venzac, Bastien et al. [21], followed by overnight immersion in isopropanol (IPA).

PDMS curing and microfluidic fabrication. PDMS silicone elastomer (Sylgard^®^ 184 Dow Chemical Co., Midland, MI, USA) and curing agent (CA) were mixed at ratios of 10:1 and 15:1 for the microchannel layer and top layer, respectively. The degas processing occurred in 1 h and was followed by 1 h of curing at 70 °C.

#### 2.2.2. Selective PVA Coating, Its Contact Angle, and Device Bonding

The PVA-coating protocol followed the study of Trantidou, Tatiana, et al. [22]. Figure 2B illustrates the steps of coating PVA onto the PDMS surface. Briefly, the PDMS substrate was rinsed through IPA, air-dried, and heated at 110 °C for 40 min. To create the selective coating on the PDMS surface, Scotch tapes were used to cover the area of the outlet. Next, these substrates were treated with oxygen plasma cleaning and immediately covered with 1% PVA solution (10 min). To finish the coating, air drying and 15 min of 110 °C heating were conducted. The wettability difference regions on the PDMS substrates were briefly checked by a drop of water and measured by ImageJ software (NIH, Baltimore, MD, USA).

The two substrates were bonded together immediately after undergoing plasma cleaning conditions at 1.9 Torr, 150 W, and 20 s.

### 2.3. Dispensed Volume and Evaporation Due to Heating

#### 2.3.1. Air Bubble Formation and Relative Dispensed Liquid Volume

We first investigated the performance of the device by demonstrating its filling behaviours with the food dyes. In detail, 15 µL of food dye was firstly input into the reaction chamber. Next, a random volume of liquid was filled at inlet 1 and gently pressed manually at the chamber to initiate the filling process. The volume of filled liquid was calculated based on the formula below:

(1) $$V_{filled}=V_{chamber\;}-V_{15\;}-V_{air\;bubbles}$$

After filling, air bubbles existed, which caused variation in the final volume of the solution inside the chamber. The formation of bubbles could be derived from dissolved air in the input solution, products of biochemical reactions inside the system, or remaining air during fabrication [23]. In this case, the bubble formation could be caused by the pressing action and pre-existing air inside the microfluidic channel. Most of the air bubbles remained spherical shape and their size was measured by using the ImageJ software. By measuring the total volume of bubbles trapped inside the chamber, we determined the filled volume of liquids/samples. SPSS Statistics 28.0 was used for statistical analysis.

#### 2.3.2. Heating Experiment and Its Relative Volume Loss

After being filled, the microdevice was placed on the 70 °C hotplate. The device was measured before and after heating. The weight difference was proportional to the percentage of volume loss during the incubation period.

### 2.4. Performance of LAMP Reaction on One-Chamber Microfluidic Device

#### 2.4.1. Running cDNA of N-Gene SARS-CoV-2 Virus Sample on a One-Chamber Finger-Actuated Microfluidic Device

This experiment is to evaluate the filling performance, its compatibility between the finger-actuated microfluidic surface and LAMP assay, and its capacity to reveal colourimetric detection results. For positive samples, the cDNA of the N-gene SARS-CoV-2 virus (Integrated DNA Technologies, Coralville, IA, USA) was used for testing the colourimetric LAMP on chip with a concentration of 5000 copies/µL. Ultrapure water (Invitrogen, Waltham, MA, USA) was used as a no-template control (NTC) or blank sample. The mixture of 2X Warmstart Colourimetric LAMP master mix and primer was pre-loaded into the chamber via inlet 2 (reagent inlet). The pre-loaded microfluidic devices were kept at 4 °C for a few hours until usage or −20 °C for 1 or 3 days until use.

To start, the sample was loaded at inlet 1 and pressed into the chamber to initiate the filling process. After filling, the devices were covered with paraffin and incubated in the 70 °C hotplate for about 30 min. The colour changing from pink to yellow indicated a positive reaction.

#### 2.4.2. Running Clinical Samples on a One-Chamber Finger-Actuated Microfluidic Device

We evaluated the performance of our one-chamber prototype using 4 positive, 1 negative, and 1 blank samples. The blank sample was the UTM solution containing no nucleic acid. Each sample was loaded into the chamber via the inlet and mixed with the pre-loaded master mix of LAMP reagent and specific primers. The chamber was heated at 65 °C until the colour change from pink to yellow occurred. The colour would remain pink in a negative test. The result was directly viewed from the chamber.

## 3. Result and Discussion

### 3.1. LAMP Assay and Clinical Samples

**Figure 4:**
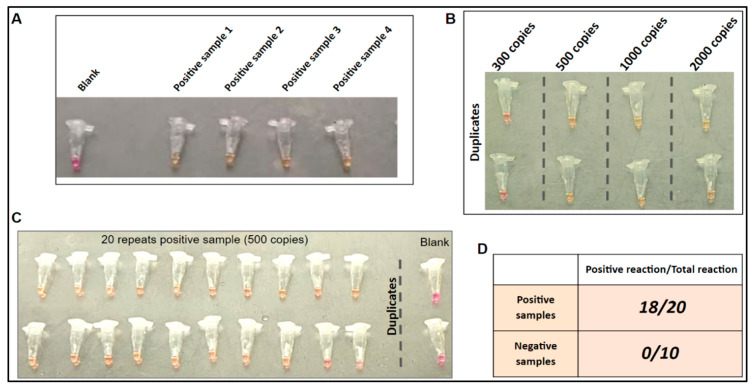
One-step LAMP on clinical samples. (**A**) LAMP on four unprocessed SARS-CoV-2 positive clinical samples to confirm positive reaction. (**B**) LOD of the one-step reaction. (**C**) LOD repeated 20 times for consistency. (**D**) One-step LAMP on 20 SARS-CoV-2 positive and 10 SARS-CoV-2 negative clinical samples.

Higher temperature for one-step LAMP for thermolysis. For concurrent thermolysis and the LAMP reaction, a high-temperature LAMP is required. To evaluate the performance of the SARS-CoV-2 LAMP assay at elevated temperatures, we conducted a high-temperature LAMP assay using RNA controls from a previous study. The LAMP reaction was effective up to 65 °C, which is five degrees higher than the previously reported working temperature [18]. This elevated temperature was subsequently employed to test four clinical samples that were confirmed to be positive for SARS-CoV-2 (Figure 4A). The LAMP assay using these samples showed positive results within 25 min, while the blank control remained negative.

Limit of detection (LOD) of LAMP reaction. As shown in Figure 4B, the results showed that the assay was able to detect viral concentrations as low as 500 copies per reaction after 25 min of incubation. Additionally, with this concentration, positive reactions were observed in 18 out of 20 replications when using positive sample 1 (Figure 4A).

Specificity on different clinical samples. As shown in Figure 4D, positive reactions were observed in 18 out of 20 positive samples, while none of the negative samples gave a positive reaction. These findings suggest that the assay is specific to the detection of SARS-CoV-2. In our previous study, the cross-reactivity for the LAMP assay was conducted by using the NATtrolTM Respiratory Panel 2 (RP2) controls (including 22 common respiratory flora and other viral pathogens) [18]. The LAMP assay was proved to be highly specific to the SARS-CoV-2 virus.

### 3.2. Finger-Actuated Microfluidic Device Fabrication and Evaluation

#### 3.2.1. Working Principle of the Finger-Actuated Microfluidic Design and Its Operation

In an isothermal amplification assay, it works based on the enzyme activity. Thus, it is important to maintain the appropriate concentrations of all reagents to promote enzyme activity. Here, this finger-actuated microfluidic device is designed to support the self-dose input volume with minimal errors caused by the variation in pipette types.

The microfluidic devices were made of PDMS, which can be deformable and obtain good optical properties for LAMP colourimetric detection. As shown in Figure 3A above, the design of a microfluidic device consists of a sample inlet (inlet 1 reservoir), a LAMP master mix inlet (inlet 2), an outlet reservoir, a reaction chamber, and three connecting channels. The inlet 1 and outlet reservoir are designed in a cylindrical shape (∅3 mm × H5 mm), which links to the reaction chamber via the connecting channels (at micro size). The channel connecting with the outlet will act as the gas release channel to allow the air displacement during the liquid filling process. The reaction chamber is also used for pressing purposes. Thus, the microfluidic device has a small and compact design to enhance its portability. Besides, it has simple operations based on pressing actions, which could be performed by many people.

In the microfluidic system, the capillary pressure is non-negligible and influential in the liquid propelling due to the contact angle of the channel’s surface and its meniscus curve at the filling front (the interface of liquid–solid–gas) [15]. In specific, the capillary pressure in the rectangular channels is calculated based on the Young–Laplace formula [24]:

(2) $$P_{c}=-\gamma [\frac{{cos}_{\theta_{t}}+{cos}_{\theta_{b}}\;}{h}+\frac{{cos}_{\theta_{l}}+{cos}_{\theta_{r}}\;}{w}]$$

where

- *P_c_* is the capillary pressure in the channel.
- γ is the surface tension of the liquid in the microchannel.
- *Ɵ_t_*, *Ɵ_b_*, *Ɵ_l_*, and *Ɵ_r_* are the contact angles (CAs) of the top, bottom, left, and right walls of the channel, respectively.
- *h* is the height, and *w* is the width of the channel.

According to Formula (2), capillary pressure depends on the channel’s contact angle (CA), its dimensions, and the surface tension of the liquid. A lower contact angle can increase the capillary pressure to promote the liquid propelling into the microfluidic liquid. However, in this case, the capillary pressure barrier still exists, which prevents the self-filling into the channel for several reasons. First, transitioning from an open space (inlet 1 reservoir) to a closed microfluidic channel (inlet 1 channel) alters the meniscus curvature at the channel’s end due to the abrupt reduction in dimensions, creating a pressure barrier that halts liquid flow. Additionally, air trapped inside the channel when filling the sample into the inlet 1 reservoir can contribute to the cause of the pressure barrier. Therefore, applying pressure to the chamber is necessary to overcome this pressure barrier and initiate liquid flow.

By gently pressing the chamber, it produces negative pressure within the microfluidic network to draw the sample from the inlet 1 reservoir to the microchannels and fill up the reaction chamber. Additionally, the inclination feature connecting the inlet channel, and the chamber is designed to prevent backward movement of liquid (due to a sudden decrease in capillary pressure). This feature also aims to guide the flow to reach the bottom of the chamber better. The sudden reduction in size and hydrophilicity of the channel surface increases the pressure barrier, and from that the filling will stop when the chamber is full. Especially, the reaction chamber has a concave-up shape, which could be beneficial to hold and concentrate liquid for colourimetric detection purposes. Furthermore, in our study, we found that a channel dimension of 200 µm is optimal for both the fabrication conditions (using a 3D printer to make the moulds) and for quickly filling the chamber. Smaller channels would take longer to fill the chamber.

**Figure 5:**
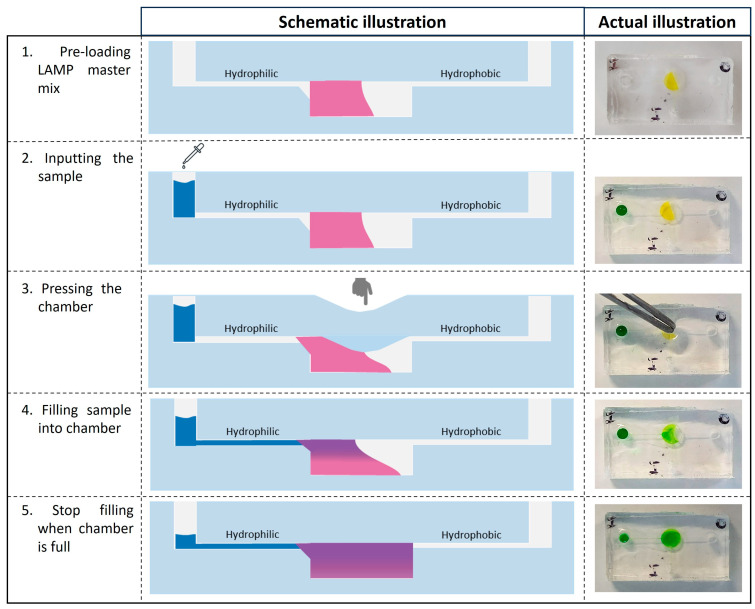
Illustration of the finger-actuated microfluidic device and workflow. 1—The LAMP 2X master mix has been preloaded inside the reaction chamber; 2—The random sample volume was dropped at the inlet. 3—The reaction chamber was slightly pressed with tweezers to initiate the fluid flow. 4—The sample has started to move to the chamber. 5—The filling would stop when the chamber was full of liquid. Note: LAMP master mix indicates as pink (schematic illustration) and yellow (actual device); Sample solution indicates as blue schematic illustration) and green (actual device).

The operation of this microfluidic device is illustrated in Figure 5. The first 15 µL of liquid or the colourimetric LAMP 2X Master Mix would be preloaded into the microfluidic device. The filled LAMP reagent must not cover the outlet channel to prevent the block of air displacement and fluid flow during the filling process. After placing the sample at inlet 1, a slight press at the chamber (Figure 5 (3)) was performed to start the filling process. The liquid flow will stop when the chamber is full (Figure 5 (5)).

In conclusion, this design for a microfluidic device offers a user-friendly operation that is based on a pressing action. In particular, this device can be used with different types of pipettes, like disposable micropipettes, exact pipettes, or micropipettes. Therefore, it holds the potential to be used in non-clinical or low-setting areas, as well as by people without laboratory training skills.

#### 3.2.2. PVA Coating and the Contact Angle of the Coated Surface

**Figure 6:**
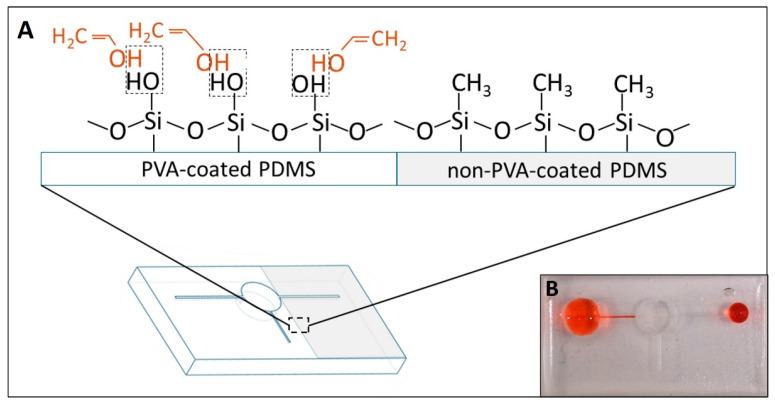
(**A**) shows the difference in molecular interaction between the PVA-coated and non-PVA-coated regions. The dashed line indicates the hydrogen bonding establishment. (**B**) shows the difference in wettability of the PDMS surface after finishing the selective coating.

To enhance the adsorption of PVA, PDMS substrates were pretreated with oxygen plasma to create the different radicals (Si–OH, C–OH and –COOH) on their surface, resulting in longer stability of the PVA absorption on the PDMS surface [22]. Also, it was reported that the oxygen plasma-treated PDMS had better adsorption of PVA onto the surface than the original PDMS [25] and maintained the stability of hydrophilicity up to 30 days after coating [22]. In specific, the –OH functional group on the oxygen plasma-treated PDMS would form the hydrogen bonding with the PVA molecule, resulting in a permanent hydrophilic surface. Therefore, taking advantage of the importance of the plasma cleaning step, we could produce the different wettability regions within one device. Specifically, the region covered by tapes before oxygen plasma could inhibit the contact and adsorption of PVA on PDMS, resulting in remaining hydrophobicity. Figure S1 illustrates the difference in the contact angle of the PDMS substrate after the PVA-coating process. The plasma-treated region had the contact angle (CA) value of PVA-coated areas around 22.32 ± 1.4° (*n* = 9), while the untreated region showed hydrophobicity with its common CA of PDMS (around 109°) [26]. In addition, Figure 6B shows that the selective PVA-coating process occurred successfully inside the channel.

The oxygen plasma cleaning targets the single bonds in the contaminant’s molecule, including C–H, C–C, and C–O, and then oxidised them into C–O, C=C and C=O, respectively. Meanwhile, the presence of many hydrogen bonds possibly decreases the efficiency of plasma cleaning [27]. Moreover, an earlier study showed that post-oxygen plasma on a PVA-coated surface can enhance stability and has the lowest hydrophilicity on other materials (poly(methyl methacrylate) (PMMA)) [28]. Importantly, the primary chemical composition and interaction chain of PVA was found to remain after the post-oxygen plasma. Therefore, in our case, we believe that the oxygen plasma cleaning on PVA-coated PDMS might not be affected significantly, as it would not destroy completely the hydrogen bonds created between PVA and PDMS molecules. On the other hand, no issue was found when PVA-coated PDMS underwent the oxygen plasma cleaning again for bonding in this study, and the fabricated devices had the same performance after storing at room temperature in a sealed Petri dish.

### 3.3. Liquid Filling, Heating Experiment and Its Efficiency

#### 3.3.1. Air Bubble Formation and Relative Dispensed Liquid Volume

We first investigated the performance of the device by demonstrating its filling behaviours with the food dyes. The filling process with water usually took around one minute (Video S1), including re-pressing the chamber to complete the process.

**Figure 7:**
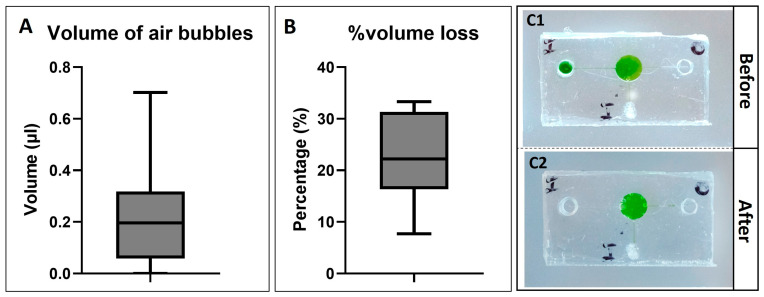
(**A**) shows the total measured volume of air bubbles trapped inside the device after the filling process (*n* = 13). (**B**) shows the percentage of volume loss after heating (*n* = 13). The microfluidic devices are shown before heating (**C1**) and after heating (**C2**).

The total measured volume of bubbles trapped inside the chamber varied, ranging from 0 to 0.705 µL (Figure 7A). According to Formula (1), the filled volume of liquid/sample that stayed inside the chamber was estimated in the range of 10.295 to 11 µL. This range was also similar to the desired amount of sample for the LAMP reactions. The variation in the inputted sample volume might cause some variation when running the amplification reactions. However, it did not affect the LAMP reactions which have been proved in the following results.

#### 3.3.2. Heating Experiments and Volume Loss

In this experiment, the filled microfluidic devices were placed in the 70 °C hotplate to mimic the incubation conditions of the LAMP reaction. Around 65–70 °C, the temperature is not as high as the one needed for a PCR reaction (~95 °C), but it still causes some potential issues that affect the LAMP reaction in the microscale, such as evaporation [29]. In specific, evaporation can cause an alteration in the concentration and ratios of different reagents in LAMP reactions. As a result, LAMP reactions can be affected, such as causing a false positive or negative due to lengthening the incubation time. In addition, when the incubation lasts longer, the volume of air bubbles inside the chamber will expand, or more gas molecules can invade the microfluidic network due to the PDMS porosity. Consequently, it also contributes to the inhibition or interference of reading out the amplified results, which is based on colour change.

Figure 7(C1,C2) illustrates the finger-actuated devices before (Figure 7(C1)) and after heating (Figure 7(C2)) at 70 °C for 25 min. Most of the liquid remained inside the reaction chambers after the heating process. The weight of the device before and after was measured to determine the volume loss due to evaporation. The percentage of volume loss was various. The maximum measured value was about 33.33%, while the minimum measured value was 7.7%. The variation in volume loss could be because of the uncertainty during the fabrication process. Furthermore, there were a few bubbles that appeared inside the chamber; however, it might not affect the visualisable ability for colourimetric detection.

In this study, the reaction chamber was designed to stay in the concave-up position, which has been proved to have better performance after the incubation period compared to the concave-down chamber (Figure S2). In specific, the concave-up chamber reduced the liquid escaping via the channels since the channel stayed above the chamber. In contrast, the concave-down chamber connecting to the channel at the bottom resulted in the liquid escaping easily due to the chaos created by the increasing temperature during the heating process. Moreover, the concave-down chamber might trap the large air bubbles inside and stay on the top surface of the microfluidic chip (Figure S2(B2)). As a result, it interfered with the visualisation of the colourimetric LAMP assay later. Interestingly, a large inlet/outlet can also work as a reservoir to contain and prevent the samples from escaping the device. As a result, the device remains hygienic and reduces the risk of contamination.

### 3.4. LAMP-on-Chip Reactions

Incorporating this streamlined LAMP-based COVID-19 testing into POC devices offers significant benefits. Eliminating sample processing reduces testing time and costs, making it particularly advantageous for resource-limited settings and also non-clinical settings, such as airports, schools, or workplaces. Herein, our one-chamber prototype also validated the reproducibility of this simplified protocol with the cDNA of the N-gene SARS-CoV-2 virus and clinical sample.

#### 3.4.1. Running SARS-CoV-2 cDNA on One-Chamber Finger-Actuated Microfluidic Prototypes

The filling process in the LAMP-on-chip reaction took longer than 2 min and required multiple pressing actions compared to when working with water (Video S2). The LAMP master mix contains several components, including low tris-buffer, polymerase, and phenol red, which slightly increases the viscosity of the LAMP master mix compared to water. It is likely that the higher viscosity of the solution increased the resistance between the liquid and microfluidic channels.

On the other hand, when pressing to initiate the sample moving into the chamber, the LAMP master mix was observed to spill into the inlet 1 channel. However, the LAMP master mix was delivered back to the chamber along with the flow of samples and caused no issue with the LAMP reactions. Besides, The inlet 1 and outlet reservoirs are designed with significant heights (approximately 5 mm) to contain samples and provide space for gas or liquid to escape during incubation, preventing device outflow for the sake of hygiene. In specific, a small amount of sample might remain in the inlet 1 reservoir, resulting in creating pressure to block the liquid in the chamber. The hydrophobic outlet channel is preferable for gas release than liquid escape due to the pressure barrier caused by the sudden increase in the contact angle. However, in our experiment, in some cases, we still observed some liquid escaping via the outlet channel, but the flow would stop at the end of the channel connected to the outlet reservoir, and there was no outflow from the devices.

**Figure 8:**
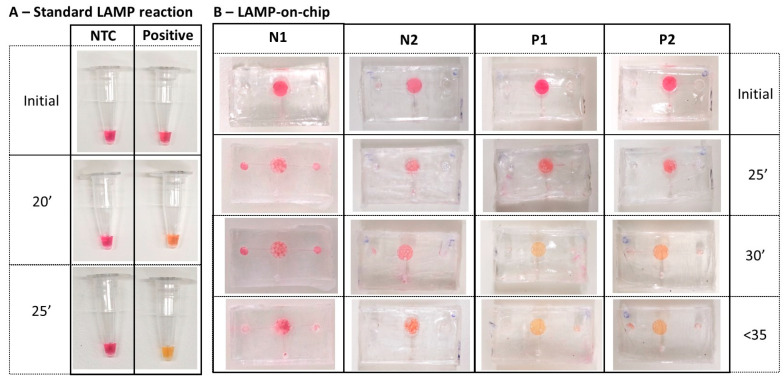
LAMP reaction under different conditions: (**A**) LAMP-on-tubes reactions; (**B**) LAMP-on-chip reactions for no-template control (N1 and N2) and positive sample (5000 copies/µL of cDNA N-gene SARS-CoV-2) (P1 and P2).

As shown in Figure 8, the LAMP-on-chip reaction produced a colour change in positive samples starting at 30 min. This indicates that the PVA coating did not inhibit the LAMP assay, and the deeper reaction chamber was beneficial for observing the colour change during incubation. Compared to our previous study, the LAMP assay was optimised for the approximately 15 µL reaction [18]. The total reaction volume for the LAMP-on-chip reaction was 13 µL, including 8 µL of master mix and 5 µL of sample. During incubation, trapped air either expanded or the volume reduced due to evaporation, making it difficult to recognise the colour change. In this study, the new design of a microfluidic device with a single chamber can contain nearly double the volume of the LAMP assay. The concentration of LAMP components was maintained the same while increasing the volume, thereby maintaining the accuracy while enhancing the colourimetric detection.

On the other hand, these reactions required longer incubation times at the same temperature. This delay may be due to several factors. First, PCR tubes are made of polypropylene (PP), which has similar thermal conductivity, but slightly higher thermal capacity compared to PDMS. Consequently, PP’s superior heat storage and transfer capabilities result in more efficient heating in LAMP-on-tube reactions. Thus, at the same temperature, LAMP-on-chip reactions may require more time to accumulate the necessary energy for incubation. Additionally, the ratio of LAMP reagents to samples varied slightly across different microfluidic devices, differing from the optimal ratio under standard conditions. This variability may contribute to the longer incubation times and the differences in colour intensity in positive samples. Nonetheless, these variations did not prevent the reaction, and the colour change was observed.

Regarding pre-storage capability, LAMP-on-chip devices were stored at −20 °C for 1 and 3 days, then thawed and used for LAMP reactions. The device performance in sample filling remained consistent after 1 and 3 days of storage. As shown in Figure S3, the 1-day stored LAMP-on-chip devices (n = 3) successfully conducted the amplification reaction, whereas the 3-day stored devices (n = 3) failed to produce a colour change in positive samples (cDNA). This inhibition in the 3-day stored devices could be attributed to prolonged exposure to trapped air inside the devices or during the transfer of LAMP. The movement of preloaded reagents could potentially block the input channels, causing more air to be trapped inside instead of displaced with the input fluid. From that, it increases the chance of mixing air in the master mix and changes pH levels, especially in small-volume samples. These reasons can contribute to the colour of LAMP being vaguer and too many bubbles appearing upon starting the incubation in both 1-day and 3-day stored devices. Additionally, when combined with primers, the LAMP master mix might decrease their stability compared to the LAMP without primers. Therefore, further investigation is necessary for long-term storage of pre-loaded LAMP on devices, including reagent separation and operation conditions. Moreover, the specific heat capacity of PDMS is low at low temperatures [30], meaning it requires more time to reach the desired temperature. Although the PDMS devices were thawed back to room temperature, it was expected that the thermal properties of PDMS could be affected slightly. Consequently, the incubation time is slightly longer due to the slight decrease in specific heat capacity after being stored at −20 °C.

#### 3.4.2. Running SARS-CoV-2 Clinical Samples on One-Chamber Finger-Actuated Microfluidic Prototypes

**Figure 9:**
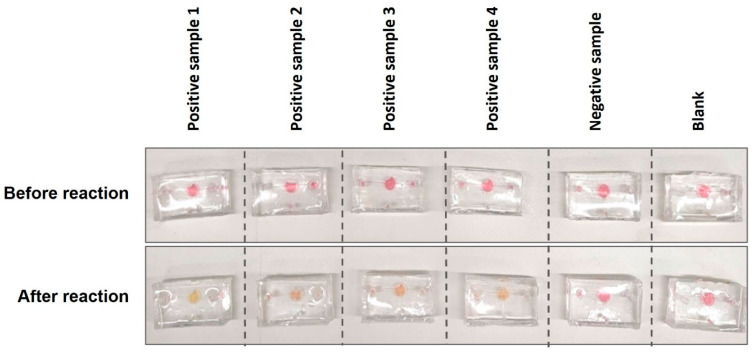
Running one-step LAMP for clinical samples on finger-actuated microfluidic devices.

The prototype was then tested on the clinical samples. As shown in Figure 9, the one-step LAMP on a microfluidic device successfully amplified four positive clinical samples, which turned yellow after 35 min of incubation. In contrast, the negative sample and blank remained pink. As previously discussed, the incubation period was longer than under normal conditions. Additionally, the intensity of the yellow colour varied among the four positive samples, likely due to differences in the viral load before and after reaching the reaction chamber.

### 3.5. Challenges and Future Directions

Challenges remain before the widespread adoption of LAMP-based COVID-19 testing in POCT devices. Optimising the assay for different sample types (e.g., UTM, saliva, blood, urine) is crucial as sensitivity and specificity may vary depending on the sample and viral load. Robust quality control measures are necessary to ensure accurate and reliable results, especially in non-clinical settings.

In the future, this integrated system of colourimetric LAMP and finger-actuated microfluidic devices require further investigation for long-term storage of LAMP on devices. In specific, it includes the separation of reaction components based on the current designs and a sealing system preventing air invasion while maintaining internal pressure. In addition, as the colourimetric LAMP master mix is highly dependent on the pH level, it is crucial to prevent its interaction with humidity and the atmosphere, especially during the pre-loading step. Thus, the optimisation of the working environment and operation should be addressed.

The relationship between colour change and sample concentration can also be investigated to provide quantitative readings. Furthermore, beyond COVID-19, this adaptable, streamlined system can potentially be used to detect other pathogens, bolstering disease surveillance and control efforts. This system would support healthcare professionals to proactively identify and combat various infectious diseases, contributing to a comprehensive and resilient approach to healthcare.

## 4. Conclusions

One-step LAMP-based testing offers potential benefits for POC devices, including faster testing times, reduced costs, and versatility in sample types. Additionally, a finger-actuated capillary-driven microfluidic device was developed to integrate with the LAMP reaction, enabling easy use through simple pressing actions This device can be used to pre-store the LAMP master mix for short-term (within a day), self-dose sample amounts, and to enhance colourimetric detection. The promising results of this study suggest that LAMP-based testing could revolutionise POC diagnostics and improve disease management.

## Figures and Tables

**Table 1:** Sequences of primers targeting the SAR-CoV-2 virus.

Primer	Sequences
F3	ACCGAAGAGCTACCAGACG
B3	TGCAGCATTGTTAGCAGGAT
FIP	TCTGGCCCAGTTCCTAGGTAGT-ATTCGTGGTGGTGACGGTA
BIP	AGACGGCATCATATGGGTTGCA-GCGGGTGCCAATGTGATC
LF	GAAATACCATCTTGGACTG
LB	CTGAGGGAGCCTTGAATACACCAA

## Data Availability

All data needed to evaluate the conclusions in the paper are present in the paper and the Supplementary Materials.

## Supplementary Materials

The following supporting information can be downloaded at: https://www.mdpi.com/article/10.3390/bios14090410/s1. Figure S1: Contact angle of the PVA-coated PDMS. Figure S2: The concave-up chamber before (A1) and after heating (A2); the concave-down chamber before (B1) and after heating (B2). Figure S3: LAMP-on chip reactions after stored at -20 °C for (A) 1 day; and (B) for 3 days. No template control (NCT) was blank/water and positive sample was 5000 copies/µl of cDNA N-gene SARS-CoV-2. The supplementary materials (video) can be downloaded at Zenodo: https://zenodo.org/records/12569549 (accessed on 27 June 2024) for video. Video S1: The filling experiment was illustrated with water-containing food dyes. Video S2: The filling experiment was illustrated with the LAMP master mix and samples.
