# Supplementary material for: A Finger-Actuated Sample-Dosing Capillary-Driven Microfluidic Device for Loop-Mediated Isothermal Amplification
Source: Biosensors (Basel). 2024 Aug 23;14(9):410. doi: 10.3390/bios14090410 (PMC11430145; doi:10.3390/bios14090410)
Supplement: Supplementary file 1 [file biosensors-14-00410-s001.zip › biosensors-3104347-supplementary.pptx]

## Slide 1
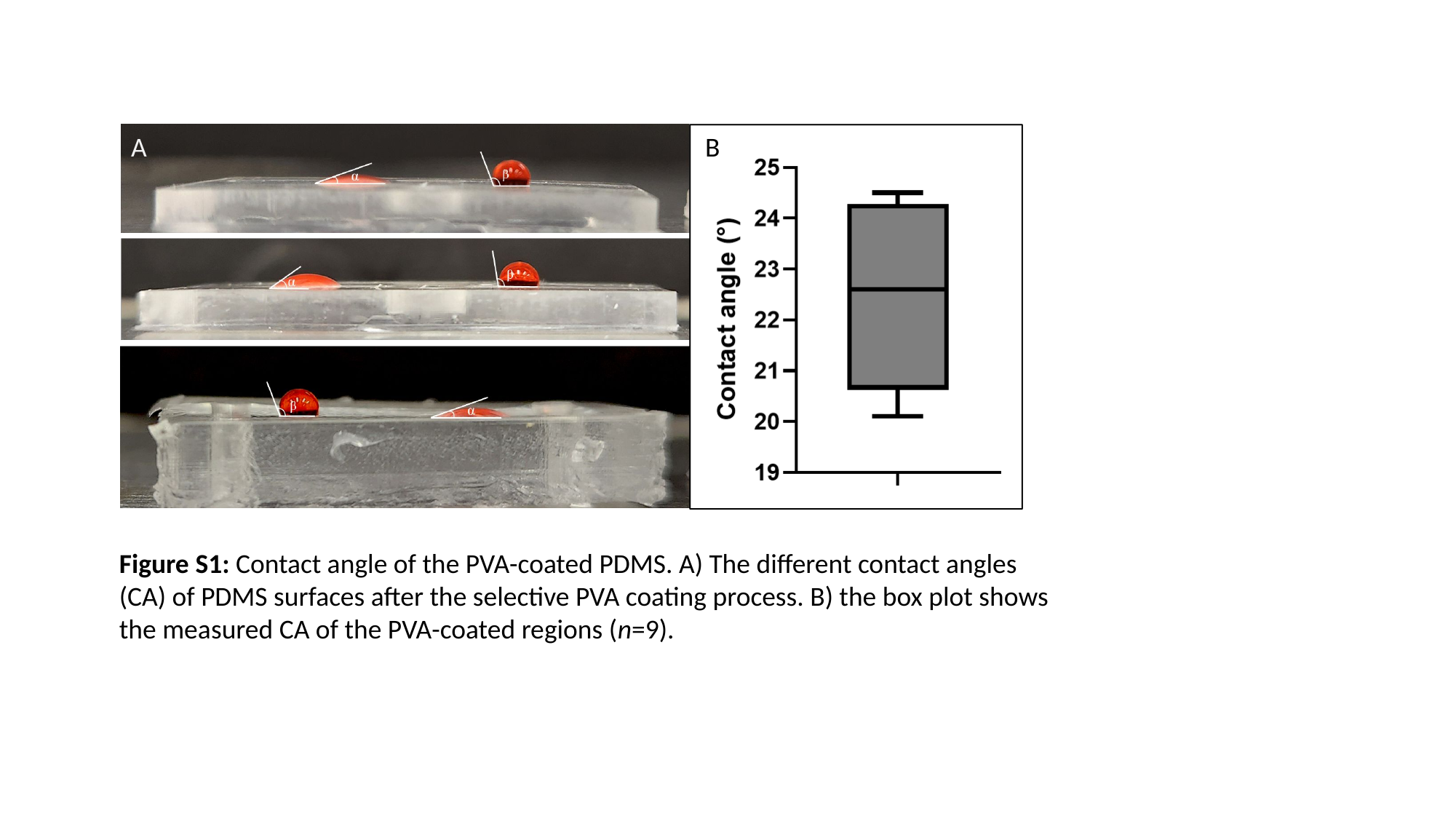

A
B
Figure S1: Contact angle of the PVA-coated PDMS. A) The different contact angles (CA) of PDMS surfaces after the selective PVA coating process. B) the box plot shows the measured CA of the PVA-coated regions (n=9).

## Slide 2
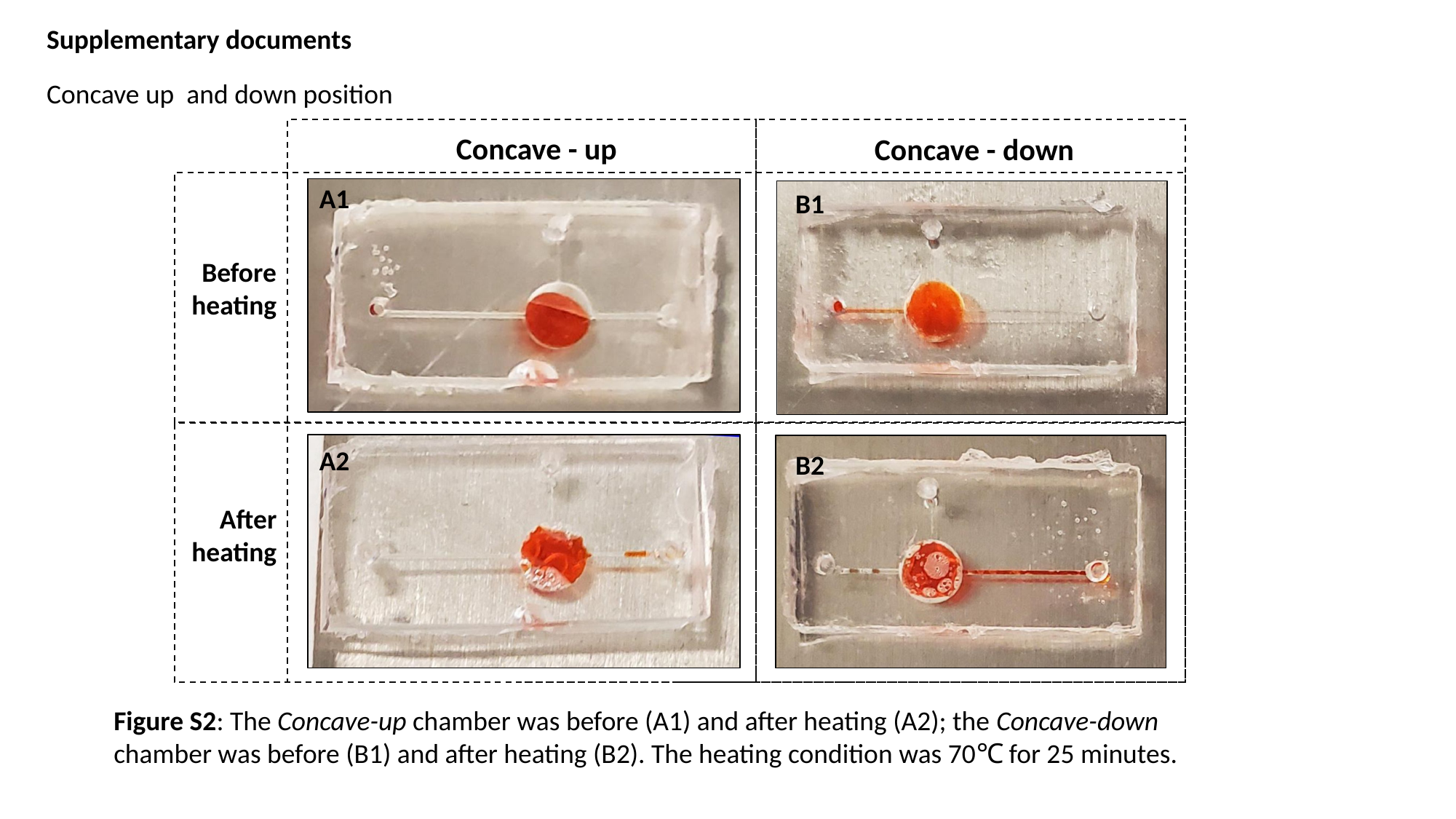

Supplementary documents
Concave up and down position
Concave - up
Concave - down
Before heating
After heating
Figure S2: The Concave-up chamber was before (A1) and after heating (A2); the Concave-down chamber was before (B1) and after heating (B2). The heating condition was 70℃ for 25 minutes.
A1
B1
A2
B2

## Slide 3
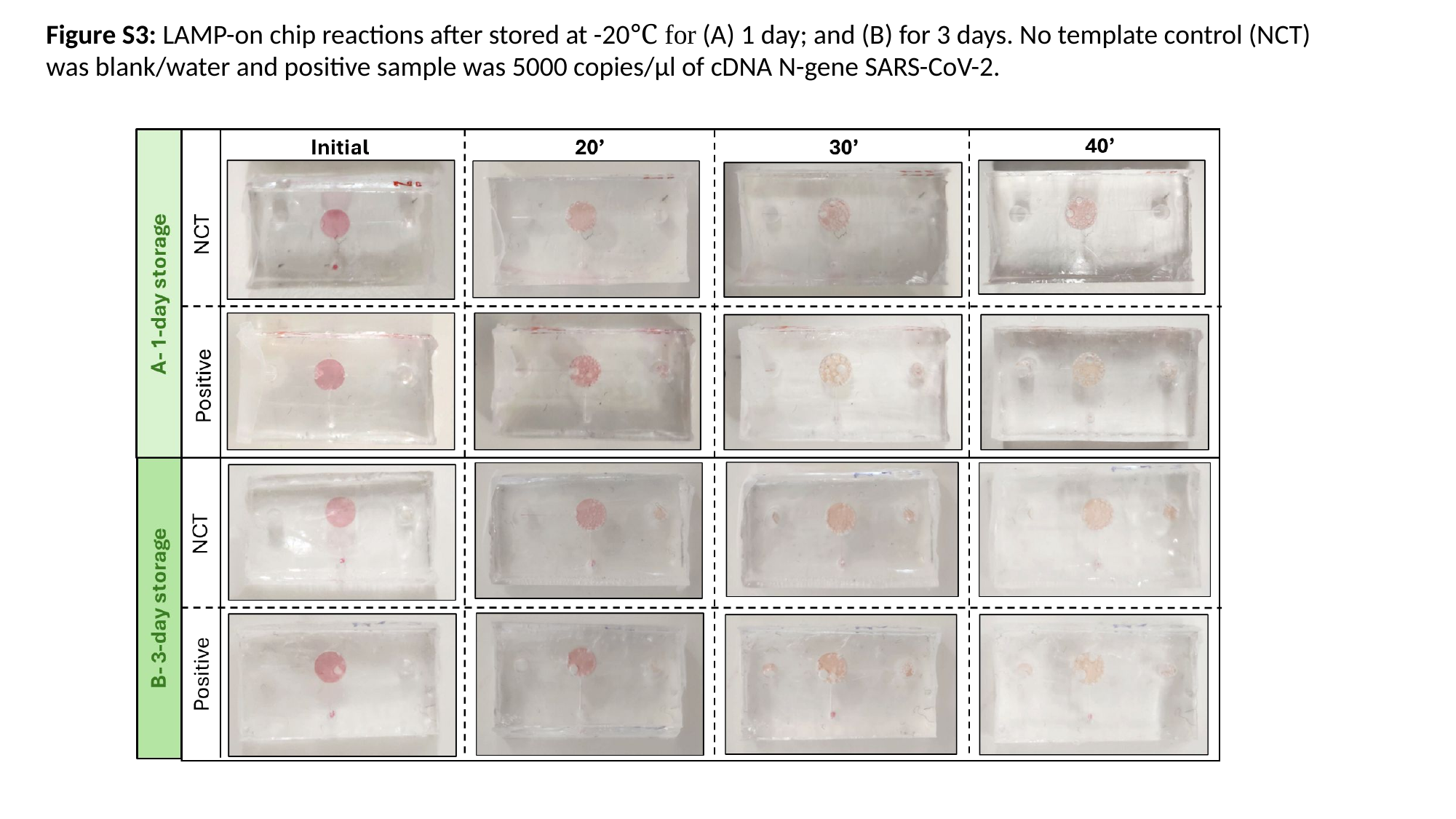

Figure S3: LAMP-on chip reactions after stored at -20℃ for (A) 1 day; and (B) for 3 days. No template control (NCT) was blank/water and positive sample was 5000 copies/µl of cDNA N-gene SARS-CoV-2.
